# Lifetime MDMA use and associations with meaning in life in the context of childhood trauma

**DOI:** 10.1038/s41598-026-37721-6

**Published:** 2026-02-10

**Authors:** Michelle Olofsson, Kasim Acar, Otto Simonsson, Maria Bragesjö, Tonya White, Rita Almeida, Predrag Petrovic, Alexander Lebedev

**Affiliations:** 1 Center for Psychiatry Research & Center for Cognitive Neuropsychiatry, Department of Clinical Neuroscience, Karolinska Institutet, Stockholm, Sweden; 2 Section on Social and Cognitive Developmental Neuroscience, National Institute of Mental Health, National Institutes of Health, Bethesda, MD USA; 3 Department of Neurobiology, Care Sciences and Society, Karolinska Institutet, Stockholm, Sweden; 4 Center for Psychiatry Research, Department of Clinical Neuroscience, Karolinska Institutet, & Stockholm Health Care Services, Stockholm, Region Stockholm Sweden; 5 Stockholm University Brain Imaging Center, Stockholm University, Stockholm, 114 18 Sweden

**Keywords:** Meaning, Purpose, Childhood, Trauma, MDMA, Psychedelics, Health care, Medical research, Psychology, Psychology, Risk factors

## Abstract

Recent research has highlighted the potential of 3,4-methylenedioxymethamphetamine (MDMA)-assisted psychotherapy for the treatment of post-traumatic stress disorder and other trauma-related psychiatric conditions. However, less is known about the effects of MDMA use on psychological well-being in populations with trauma histories outside of clinical contexts. In the present study, we assessed associations between naturalistic lifetime MDMA use (i.e., having ever used MDMA) and an overlooked psychological predictor of resilience – meaning in life. Using data from a survey study of a sample of Swedish adults (*n* = 807), multiple linear regression models were fit to assess associations between lifetime MDMA use and meaning in life, as well as interactions with childhood trauma history. Meaning in life was measured using the presence subscale of the 10-item meaning in life questionnaire (MLQ). While lifetime MDMA use was not significantly associated with meaning in life overall (β = 1.80, *p* = 0.055), a significant interaction emerged between lifetime MDMA use and childhood trauma on meaning in life (β = 4.06, *p* = 0.012). Specifically, MDMA use was associated with higher meaning in life among individuals with a history of childhood trauma. Together, these associations warrant further longitudinal and experimental research to test whether MDMA exposure relates to changes in meaning in life.

**Supplementary Information:**

The online version contains supplementary material available at 10.1038/s41598-026-37721-6.

## Introduction

 Despite existing treatment options, psychological trauma remains a pervasive global mental health issue, as recognized by the World Health Organization^1^. Trauma-related conditions, such as post-traumatic stress disorder (PTSD), are difficult to treat, with some research suggesting only 50% of individuals respond to first-line psychotherapeutic and pharmacological interventions^2,3^. Moreover, many patients experience recurring symptoms, underscoring the need for more effective therapies to address the complex and persistent reactions to trauma. Recently, a resurgence in research on 3,4-Methylenedioxymethamphetamine (MDMA)-assisted psychotherapy (MDMA-AT) has offered promise as one of the few potential new pharmacological treatments for trauma^4–6^. Evidence from a completed phase 3 clinical trial in the United States has demonstrated significant reductions in PTSD symptomatology for MDMA-AT compared to placebo^7,8^. While MDMA-assisted therapy has already been approved for clinical use in certain countries, such as Australia, the intervention was recently rejected by the Food and Drug Administration (FDA), citing the need for further research and validation^9,10^.

MDMA, often referred to in naturalistic settings as “ecstasy” or “molly”, is a synthetic amphetamine derivative known for its prosocial effects such as enhanced empathy, energy, and pleasure. Sharing properties with both psychedelic and stimulant substances, MDMA’s primary pharmacological targets include the serotonin, dopamine, and noradrenaline systems^11,12^. Clinical research on MDMA has focused primarily on MDMA as a psychotherapeutic agent alongside therapy for PTSD. To date, there have been eight published randomized clinical trials on MDMA-AT involving nearly 300 study participants, which have demonstrated promising effect sizes in the reduction of PTSD symptoms^4^. Despite growing research on the psychotherapeutic applications of MDMA-AT, the therapeutic mechanisms underlying its efficacy remain unclear. One line of evidence suggests MDMA-AT functions by dampening the fear response, thereby facilitating trauma survivors to more readily revisit and reconsolidate difficult memories^12–16^. Alternatively, it has been suggested that posttraumatic growth (PTG) may be central to the therapeutic mechanism of action, indicated by positive changes in self-perception, interpersonal relationships, or philosophy of life after MDMA-AT^17^.

One potentially related psychological mechanism of MDMA-AT that remains underexplored is the enhancement of meaning in life. Not to be confused with the meaning *of* life, meaning in life generally refers to a personal sense of purpose, coherence, or significance in one’s existence^18,19^. Perhaps most famously described by Holocaust survivor and psychiatrist Viktor Frankl in his 1964 work *Man’s Search For Meaning*, the human drive to create meaning has been proposed as fundamental to psychological resilience and overcoming extreme adversity^20^. Contemporary psychological research has since empirically supported this notion, linking self-reported meaning in life with many important factors of well-being, such as reduced depression, anxiety, and suicidality^21,22^. Recently, there has been growing research attention to the potential heightened importance of targeting meaning in life in clinical interventions for individuals with trauma-related psychiatric disorders^23^. According to the ‘meaning-making model’ of trauma, to overcome psychological stress and suffering, trauma survivors must resolve the mismatch between their ‘global’ meaning model of the world before the trauma, and the ‘situational’ meaning that a traumatic event poses^24,25^. In other words, in the aftermath of a trauma, survivors often feel as though they are thrown into a world that is unpredictable and incomprehensible to the world they once understood^26^. As such, an increasing number of studies have focused on expanding scientific inquiry on psychiatric interventions rooted in meaning-making in order to better promote long-term mental resilience and trauma recovery^27,28^.

Despite the importance of meaning-making in trauma recovery and the potential applications of MDMA-AT in the treatment of trauma-related psychiatric disorders, there exists limited investigation of the potential relationship between MDMA-AT and meaning in life. In one small survey study, 19 out of 21 participants who had previously participated in MDMA-AT reported feeling greater meaning in life as a result of the treatment^29^. Similarly, another interview study found that MDMA-AT participants described benefits beyond PTSD symptom reduction, including enhanced quality of life, improved relationships, and making new meaning of their traumatic experiences^30^.

Although there is currently little empirical work that has assessed the link between MDMA use and meaning in life, the hypothesis that MDMA use may lead to increases in meaning may be supported by literature on classical psychedelics, such as lysergic acid diethylamide (LSD) or psilocybin. A notable body of scholarly discussion has proposed that classical psychedelics have ‘meaning-enhancing properties’ that may be key to their psychotherapeutic potential^31–33^. This has been supported by clinical and naturalistic research, which has demonstrated increases in meaning in life following psychedelic use in both clinical trials and real-world settings^34–38^. While MDMA and classical psychedelics have key differences, the substances also share potentially relevant phenomenological and neurobiological effects^12^. For instance, both substance classes have demonstrated effects on social neuroplasticity and prosocial behaviors, such as alterations in self-image and social reward processing^39–42^. Considering that a stable sense of self-concept and social connection are strong contributors to meaning in life^43,44^, it is worth considering to what extent the ‘meaning-enhancing hypothesis’ of classical psychedelics applies to MDMA.

In sum, the precise therapeutic mechanisms underlying the efficacy of MDMA-AT have yet to be elucidated, and the potential link between MDMA and enhanced meaning in life may be an underexplored psychological pathway. In line with previous studies that have looked at associations between naturalistic use of classical psychedelics and meaning in life, we leveraged data from non-clinal use of MDMA^37^. While it should be noted that such associations cannot directly implicate causal relationships between MDMA use or MDMA-AT and meaning in life, naturalistic datasets offer an opportunity to study associations that are challenging to address in clinical or experimental research, as they are typically limited to small sample sizes. Given that an estimated 2.2% of young adults in the EU and 0.9% of adults in the US have reported MDMA use in the past year, neglecting to examine such associations would represent a substantial oversight of natural observational data^45,46^. While MDMA use in naturalistic settings holds known risks^4^, especially in comparison to pharmaceutical-grade MDMA used in clinical settings, observational research has also found relationships with positive outcomes^47,48^. For instance, studies have found associations between MDMA use and lower odds of major depressive episodes and perceived social-emotional benefits^47–49^.

In the present study, we employed data from a cohort of people in Sweden who have reported MDMA use. Using the Meaning in Life Questionnaire (MLQ) as an instrument to measure the presence of meaning in life, we assessed two primary hypotheses. First, we hypothesized that lifetime use of MDMA would be associated with a higher presence of meaning in life when adjusted for use of other commonly used recreational substances. Second, we hypothesized that the association between MDMA use and meaning in life would be higher among those with a history of trauma. As trauma history is often linked to lower meaning in life, we reason that if MDMA use is associated with meaning in life, the potential for an increase in meaning in life would be greater amongst traumatized individuals.

## Methods

### Sample

The present study is part of a larger longitudinal project from 2018 to 2020, where we aimed to investigate behavior and cognition related to delusion proneness and use of psychedelics. So far, four studies have been published based on this sample^50–53^. Data collection was approved by the Swedish Ethical Board (Regionala Etikprövningsnämnden i Stockholm, DNR: 2018/1040-31), and the Department of Clinical Neuroscience at Karolinska Insititutet. Informed electronic consent was also obtained from all participants prior to the procedure. All methods were performed in accordance with the relevant guidelines and adhered to the principles of the Decleration of Helsinki. The study was conducted in a population of adults in Sweden, and although the project has longitudinal components, the present study involved data from only one time-point. The initial sample size (*n* = 1032) was calculated using G*Power v3 for small and medium to large effect sizes. Participants were recruited from a combination of sources, including web-based announcements through the university platform, general participant recruitment platforms, and via social media services (i.e., Facebook and Reddit). The study was advertised as investigating the relationship between personality, learning, and beliefs (see Supplemental Materials for more details on advertising materials and participant recruitment sources). Subjects recruited through social media were intentionally recruited from forums that were expected to include the target population (i.e., discussing scientific and recreational use of psychedelics, drug policy, or substance-related issues).

The data for the present study were sampled in 2018, and out of 1032 studied subjects that were included initially, 807 completed all the questions that were used in the present study. For details on the recruitment origin of participants in the present study, see the supplemental materials (Table S1). As a part of the initial survey screening, we assessed psychiatric diagnoses and problems (including schizophrenia or other psychotic disorder, bipolar disorder, depression, childhood trauma, and OCD) and neurological diagnoses (epilepsy, head trauma, or “other”) as well as alcohol and drug use (including type of drug). In addition, socioeconomic data and information on personality and psychiatric traits were collected during the screening procedure. For the present study, we had no exclusion criteria.

### Measures

Childhood trauma history represents one of the primary independent variables in this study and was assessed through a series of two questions. The first question asked ‘Have you been psychologically traumatized as a child (< 17 years)?’. Response options included ‘No’, ‘Maybe’, or ‘Yes’. This question was intended to capture participants’ subjective experience of trauma, allowing for individual recognition of psychological distress, regardless of whether the event met formal criteria for a potentially traumatic experience. To gain further insight into the intensity and impact of the trauma, follow-up questions inquired about how frequently the participant thought about these events, how much they affected their daily life, and whether they had sought support or treatment. Following the subjective question, participants were asked to report specific ACEs from a predefined list based on the 11-item ACE questionnaire^54^. The categories included: household dysfunction (e.g., parental separation, substance abuse), neglect (emotional and physical), physical abuse, sexual abuse, bullying, and other. Our adapted questionnaire broadens the scope of the 11-item ACE-Q to include bullying and personally reported ‘other’ forms of early life trauma.

In our main analysis, we focused on participants’ subjective reports of being psychologically traumatized (‘No’, ‘Maybe’, ‘Yes’) rather than the number of ACEs, as we were primarily interested in how traumatic experiences affected the individuals. It is important to acknowledge that there are several limitations in this methodological choice. First, the use of non-validated questionnaires is subject to unknown validity and reliability^55^. Second, the use of one-item measures has unique limitations, such as higher uncertainty in reliability and lower granularity^56^. Despite these limitations, we reasoned that the subjective measure of trauma was more relevant than ACEs to the present research question. In other words, it is likely that an individual’s self-reported perception of being traumatized reflects not only the occurrence of adverse events but also the personal meaning and impact these experiences hold. This aligns with established literature, which suggests that subjective appraisal and the impact of adverse events better predict clinical outcomes^57^. To ensure transparency and maintain comparability with previous research, we also performed sensitivity analyses using ACEs as a proxy for childhood trauma.

Lifetime MDMA use represents the second primary independent variable. Participants were also asked to report their lifetime use of various other substances, including alcohol, cannabis, MDMA, opiates (e.g., heroin, morphine, opium), psychedelics (e.g., LSD, magic mushrooms/psilocybin, ayahuasca/DMT), stimulants (e.g., amphetamine, ephedrine, cocaine), and tobacco. Participants who reported having ever used a substance were coded as positive for lifetime use, and those who did not were coded as negative. It is important to note that data on frequency, dosage, and context of substance use (e.g., recreational vs. therapeutic) were not available.

The primary dependent variable of interest in this study was meaning in life. This was assessed using the Meaning in Life Questionnaire (MLQ), a frequently used instrument for measuring meaning in life, which has growing international popularity^58,59^. The questionnaire, containing 10 items and a 7-point Likert scale, is divided into two parts: the Presence of Meaning (MLQ-P; e.g., ‘I understand my life’s meaning’) and the Search for Meaning (MLQ-S; e.g., ‘I am searching for. A purpose or mission for my life’). The total individual scores for MLQ-Presence and MLQ-Search range from 0 to 35, with higher scores representing higher levels of presence and search for meaning, respectively. For the purpose of this study, we were primarily interested in the MLQ-Presence sub-score.

### Hypotheses

#### **Hypothesis 1**

Lifetime MDMA use will show a positive association with presence of meaning in life.

#### **Hypothesis 2**

Lifetime MDMA use will show an interaction with a history of childhood trauma, wherein those who have experienced childhood trauma will show a stronger association between lifetime MDMA use and presence of meaning in life.

### Statistical analysis

Before conducting any statistical analyses, an initial inspection of the data was performed. For cases where data was missing (which was only the education variable), multiple imputation using predictive mean matching was performed using the MICE package in R^60^. A total of 6 imputations and 50 iterations were run. Convergence was checked through visual inspection of trace plots (Figure S1). A single imputed dataset was extracted for further data analysis. As a sensitivity analysis, a listwise deletion approach was taken where rows with missing education data (*n* = 46) were removed. The main analyses were reproduced on the dataset with listwise deletions.

Group-level chi-square analyses were first conducted to compare those with and without childhood trauma, as well as those who have and have not reported MDMA use. To assess the relationship between childhood trauma history and meaning in life, a one-way ANOVA analysis was performed. Post-hoc tests were employed for validation when applicable.

For the main analyses, several multiple linear regression models on the primary outcome variable of MLQ-Presence, using ‘lm’ in R. To test hypothesis 1, a multiple linear regression model was fit where lifetime MDMA use was the main independent variable and MLQ-Presence the dependent variable. For hypothesis 2, an interaction model on the dependent variable of MLQ-Presence was fit, including an interaction term between childhood trauma (‘No’, ‘Maybe’, ‘Yes’) and lifetime MDMA use. All models were adjusted for the covariates of age, gender (male, female, other), educational attainment (measured in years post-high school), and lifetime use of other substances (psychedelics, alcohol, cannabis, opiates, stimulants, tobacco). These covariates were selected based on prior research, which has demonstrated associations between these demographic factors, substance use, and meaning in life^61,62^. Normality of residuals was assessed using a combination of histograms and QQ-plots. In addition, the performance library was used to visually inspect multicollinearity, homoscedasticity, and influential points^63^.

Further, exploratory analyses were conducted to investigate potential interactions between other substances of use and childhood trauma on MLQ-Presence. Each of these models applied the same covariates as in the main analyses. It is important to note that these models were exploratory in nature and thus do not hold inferential weight in this study.

All data analysis was performed in R version 4.3.2^64^.

## Results

### Sample characteristics

Table 1 provides descriptive statistics of the total study population, as well as a breakdown by lifetime use of MDMA. The participants in the total sample were predominantly female (76.5%), younger adults (Mean = 27.1, SD = 5.9), and of an educated background (Mean = 3.30 years post-high school, SD = 2.91). 225 (27.9%) participants reported a history of a psychiatric diagnosis, with major depression the most commonly reported disorder (*n* = 111,13.8%). Of all lifetime substance use rates, alcohol was the most used substance (92.4%), followed by tobacco (68.3%), cannabis (48.8%), stimulants (23.5%), MDMA (21.1%), psychedelics (17.5%), and opiates (7.8%). Notably, 6.4% of the population reported no history of substance use. The group-level comparisons by lifetime MDMA use revealed differences between participants who have used MDMA (*n* = 170) and participants who have not (*n* = 637) by age (*p* = 0.022), gender (*p* < 0.001), history of childhood trauma (*p* = 0.030), number of aces (*p* = 0.032), sexual abuse (*p* = 0.018), lifetime use of other substances (*p* < 0.001), and self-reported history of psychiatric diagnoses of ADHD (*p* = 0.020), ASD (*p* = 0.038), and major depression (*p* = 0.024).

**Table 1:** Sample characteristics stratified by MDMA use. Group comparison of demographic and psychiatric characteristics stratified by reported history of MDMA use. Statistical significance was assessed using the Wilcoxon rank sum test for continuous variables, fisher’s exact test for small sample sizes, and pearson’s Chi-squared test for other categorical comparisons.

Characteristic	*N* = 807^1^	Have not used MDMA *N* = 637^1^	Have used MDMA *N* = 170^1^	*p*-value^2^
Age	27.1 (5.9)	26.9 (6.0)	27.9 (5.5)	**0.022**
Gender				**< 0.001**
Female	617 (76.5%)	510 (80.1%)	107 (62.9%)	
Male	180 (22.3%)	119 (18.7%)	61 (35.9%)	
Other	10 (1.2%)	8 (1.3%)	2 (1.2%)	
Education^a^	3.30 (2.91)	3.39 (3.04)	2.95 (2.35)	0.17
MLQ				
MLQ-presence	19 (7)	19 (7)	19 (8)	0.67
MLQ-search	23 (8)	23 (8)	24 (8)	0.23
Self-reported psychiatric diagnosis	225 (27.9%)	168 (26.4%)	57 (33.5%)	0.068
Major depression	111 (13.8%)	89 (14.0%)	22 (12.9%)	**0.024**
Bipolar disorder	14 (1.7%)	10 (1.6%)	4 (2.4%)	0.17
Schizophrenia	0 (0.0%)	0 (0.0%)	0 (0.0%)	0.068
ADHD	57 (7.1%)	37 (5.8%)	20 (11.8%)	**0.020**
ASD	21 (2.6%)	19 (3.0%)	2 (1.2%)	**0.038**
OCD	11 (1.4%)	9 (1.4%)	2 (1.2%)	0.15
Other psychiatric disorder	86 (10.7%)	68 (10.7%)	18 (10.6%)	0.080
Childhood trauma				**0.030**
Maybe	167 (20.7%)	122 (19.2%)	45 (26.5%)	
No	487 (60.3%)	399 (62.6%)	88 (51.8%)	
Yes	153 (19.0%)	116 (18.2%)	37 (21.8%)	
Number of ACEs	0.62 (0.99)	0.59 (0.98)	0.74 (1.04)	**0.032**
Types of ACEs				
Sexual abuse	59 (7.3%)	49 (7.7%)	10 (5.9%)	**0.018**
Physical abuse	37 (4.6%)	28 (4.4%)	9 (5.3%)	0.078
Neglect	37 (4.6%)	26 (4.1%)	11 (6.5%)	0.061
Bullying	129 (16.0%)	99 (15.5%)	30 (17.6%)	0.062
Household dysfunction	200 (24.8%)	146 (22.9%)	54 (31.8%)	**0.045**
Other	41 (5.1%)	29 (4.6%)	12 (7.1%)	0.062
Lifetime alcohol use	746 (92.4%)	581 (91.2%)	165 (97.1%)	**0.010**
Lifetime tobacco use	551 (68.3%)	397 (62.3%)	154 (90.6%)	**< 0.001**
Lifetime cannabis use	394 (48.8%)	244 (38.3%)	150 (88.2%)	**< 0.001**
Lifetime stimulants use	190 (23.5%)	56 (8.8%)	134 (78.8%)	**< 0.001**
Lifetime opiates use	63 (7.8%)	21 (3.3%)	42 (24.7%)	**< 0.001**
Lifetime psychedelics use	141 (17.5%)	40 (6.3%)	101 (59.4%)	**< 0.001**
No substance use	52 (6.4%)	52 (8.2%)	0 (0.0%)	**< 0.001**

^a^Years post-high school

^1^Mean (SD); n (%)

^2^Wilcoxon rank sum test; Fisher’s exact test; Pearson’s Chi-squared test.

When asked if participants had experienced childhood trauma, 40.0% (*n* = 320) reported that they were ‘Yes’ (n = 153) or ‘Maybe’ (n = 167) traumatized as a child. A stratified summary of participants by reported history of childhood trauma can be found in Table 2. Of those who responded ‘Yes’, individuals reported an average of 1.84 (SD = 1.12) ACEs, with the most common experience reported being household dysfunction (69.3%), followed by bullying (51.6%), sexual abuse (22.2%), physical abuse (18.3%), neglect (14.4%), and other (7.8%). Those who responded ‘Maybe’ reported an average of 1.28 (SD = 0.85) ACEs, with the most common trauma being household dysfunction (55.1%). Notably, some participants reported experiencing an ACE but did not report childhood trauma. These individuals may have experienced an ACE but did not perceive it as psychologically traumatizing during childhood. A group-level chi-square comparison by trauma (‘No’, ‘Maybe’, ‘Yes’) revealed significantly different rates of psychiatric diagnosis (p < 0.001), gender distribution (p = 0.013), MDMA use (p = 0.030), psychedelic use (p < 0.001), and cannabis use (p = 0.015). A Kruskal-Wallis rank sum test revealed differences among MLQ-presence (p = 0.003) and education years post-high school (p = 0.019).

**Table 2:** Sample characteristics stratified by childhood trauma. Group comparison of demographic and psychiatric characteristics stratified by reported history of childhood trauma (‘No’, ‘Maybe’, ‘Yes’). Statistical significance was assessed using the Kruskal-Wallis rank sum test for continuous variables, Fisher’s exact test for small sample sizes, and Pearson’s Chi-squared test for other categorical comparisons.

Characteristic	No *N* = 487^1^	Maybe *N* = 167^1^	Yes *N* = 153^1^	*p*-value^2^
Age	27.0 (6.1)	27.5 (5.9)	27.0 (5.4)	0.57
Gender				**0.013**
Female	367 (75.4%)	120 (71.9%)	130 (85.0%)	
Male	116 (23.8%)	42 (25.1%)	22 (14.4%)	
Other	4 (0.8%)	5 (3.0%)	1 (0.7%)	
Education^a^	3.42 (3.17)	3.49 (2.64)	2.69 (2.17)	**0.019**
MLQ				
MLQ-presence	20 (7)	18 (7)	18 (8)	**0.003**
MLQ-search	23 (8)	24 (7)	24 (7)	0.15
Self-reported psychiatric diagnosis	86 (17.7%)	54 (32.3%)	85 (55.6%)	**< 0.001**
Major depression	32 (6.6%)	31 (18.6%)	48 (31.4%)	**< 0.001**
Bipolar disorder	4 (0.8%)	2 (1.2%)	8 (5.2%)	
Schizophrenia	0 (0.0%)	0 (0.0%)	0 (0.0%)	**< 0.001**
ADHD	29 (6.0%)	12 (7.2%)	16 (10.5%)	**< 0.001**
ASD	5 (1.0%)	6 (3.6%)	10 (6.5%)	
OCD	6 (1.2%)	1 (0.6%)	4 (2.6%)	
Other psychiatric disorder	31 (6.4%)	22 (13.2%)	33 (21.6%)	**< 0.001**
Number of ACEs	0.02 (0.15)	1.28 (0.85)	1.84 (1.12)	**< 0.001**
Types of ACEs				
Sexual abuse	0 (0.0%)	25 (15.0%)	34 (22.2%)	**< 0.001**
Physical abuse	1 (0.2%)	8 (4.8%)	28 (18.3%)	**< 0.001**
Neglect	1 (0.2%)	14 (8.4%)	22 (14.4%)	**< 0.001**
Bullying	0 (0.0%)	50 (29.9%)	79 (51.6%)	**< 0.001**
Household dysfunction	2 (0.4%)	92 (55.1%)	106 (69.3%)	**< 0.001**
Other	5 (1.0%)	24 (14.4%)	12 (7.8%)	**< 0.001**
Lifetime MDMA use	88 (18.1%)	45 (26.9%)	37 (24.2%)	**0.030**
Lifetime psychedelics use	67 (13.8%)	45 (26.9%)	29 (19.0%)	**< 0.001**
Lifetime alcohol use	443 (91.0%)	159 (95.2%)	144 (94.1%)	0.14
Lifetime tobacco use	320 (65.7%)	120 (71.9%)	111 (72.5%)	0.15
Lifetime opiates use	29 (6.0%)	18 (10.8%)	16 (10.5%)	0.053
Lifetime stimulants use	103 (21.1%)	46 (27.5%)	41 (26.8%)	0.14
Lifetime cannabis use	223 (45.8%)	98 (58.7%)	73 (47.7%)	**0.015**
No substance use	38 (7.8%)	7 (4.2%)	7 (4.6%)	0.15

^a^Years post-high school

^1^Mean (SD); n (%)

^2^Kruskal-Wallis rank sum test; Pearson’s Chi-squared test; Fisher’s exact test.

### Meaning in life scores and childhood trauma

A one-way ANOVA revealed a statistically significant difference in MLQ-Presence scores across different levels of childhood trauma (‘No’, ‘Maybe’, ‘Yes’), F(2,804) = 5.898, p = 0.003. The ANOVA for MLQ-Search scores showed a non-significant difference across the groups (‘No’, ‘Maybe’, ‘Yes’), (F(2,804) = 2.826, p = 0.060). Post-hoc pairwise t-tests further showed that for MLQ-Presence, the ‘Yes’ group (Mean = 17.80, SD = 7.77) had significantly lower scores compared to the ‘No’ group (Mean = 19.81, SD = 7.22; p = 0.003). Additionally, there was a significant difference between the ‘Maybe’ group (Mean = 18.20, SD = 7.32) and the ‘No’ group (Mean = 19.81, SD = 7.22; p = 0.015). In other words, participants who reported being ‘Yes’ or ‘Maybe’ psychologically traumatized during childhood had, on average, significantly lower MLQ-Presence scores compared to those who reported ‘No’ trauma in childhood. The pairwise t-test for MLQ-search revealed a significant difference only between the ‘Yes’ (Mean = 24.04, SD = 6.82) and ‘No’ groups (Mean = 22.55, SD = 8.02, p = 0.035). In addition, a linear regression model assessing interactions between MLQ-Search and childhood trauma history on MLQ-Presence revealed that the interaction effect between childhood trauma and the meaning search-presence axis depends on childhood trauma history (β=-0.20, 95% CI [-0.33,-0.07], p=0.004). Since childhood trauma was treated as an ordered factor (‘No’, ’Maybe’, ‘Yes’), polynomial contrasts were also applied, although the quadratic interaction was not significant (p = 0.803). To illustrate this interaction association, a Spearman correlation revealed that the strength of the negative correlation between search and presence of meaning in life increased with the severity of trauma (‘No’: r = -0.013, p = 0.767, ‘Maybe’: r=-0.115, p = 0.140, ‘Yes’: r=-0.213, p = 0.008). This suggests that as trauma levels increase, the negative association between the search for meaning and the presence of meaning becomes more pronounced. In other words, a high search for meaning has a stronger correlation with lower MLQ-Presence scores when adults have a history of childhood trauma.

**Fig. 1:**
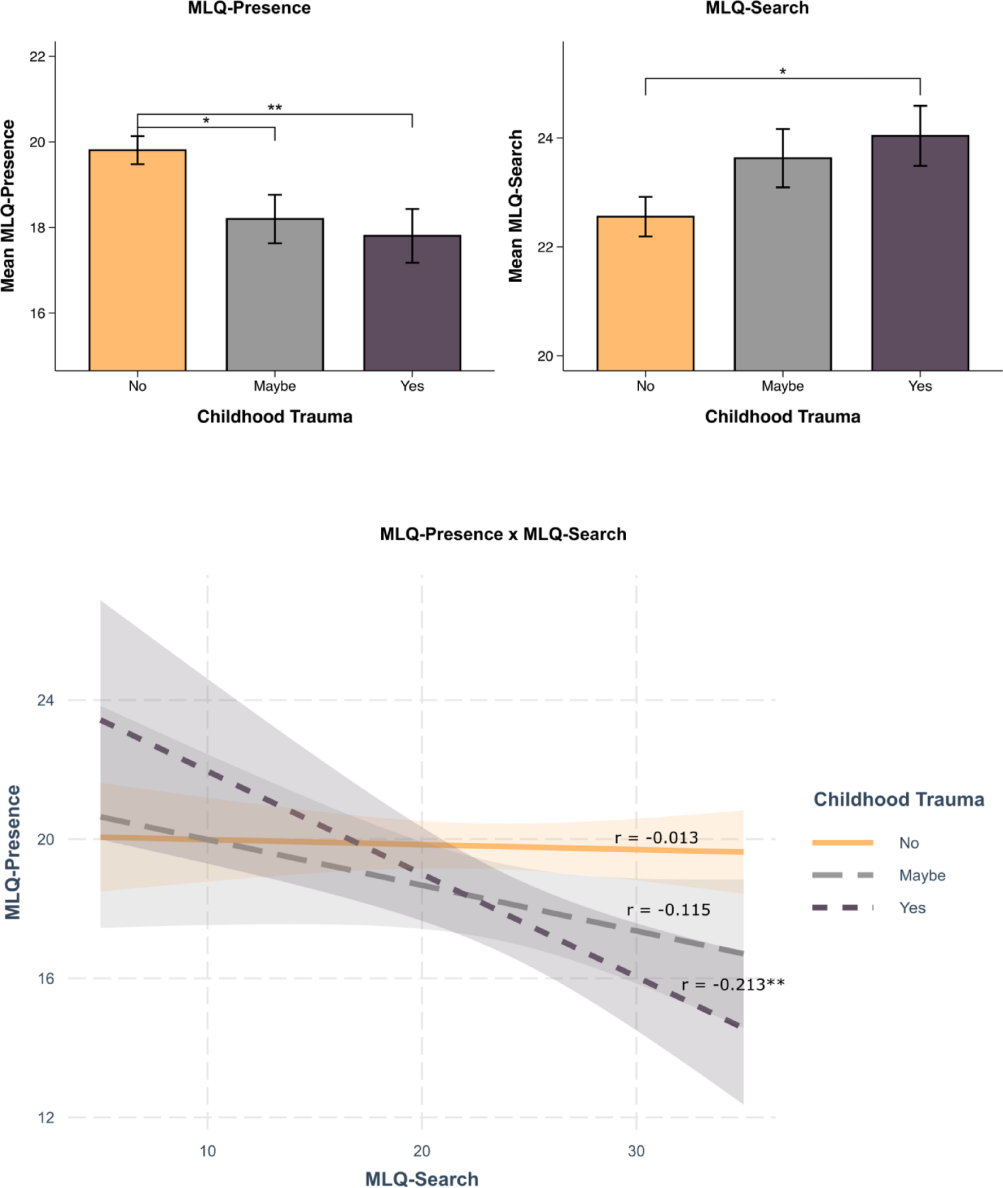
Meaning in life and childhood trauma. (**A**) Bar chart displaying the mean MLQ-Presence scores across three levels of psychological trauma: ‘No’, ‘Maybe’, and ‘Yes’. (**B**) Bar chart showing the mean MLQ-Search scores grouped by the same levels of psychological trauma. (**C**) Interaction plot illustrating the moderating effect of psychological trauma level (‘No’, ‘Maybe’, ‘Yes’) on the linear relationship between MLQ-Search and MLQ-Presence scores. Significance levels are indicated by the following symbols: * (p < 0.05), ** (p < 0.01), based on pairwise t-tests.

### Hypothesis 1: meaning in life and lifetime MDMA use

**Fig. 2:**
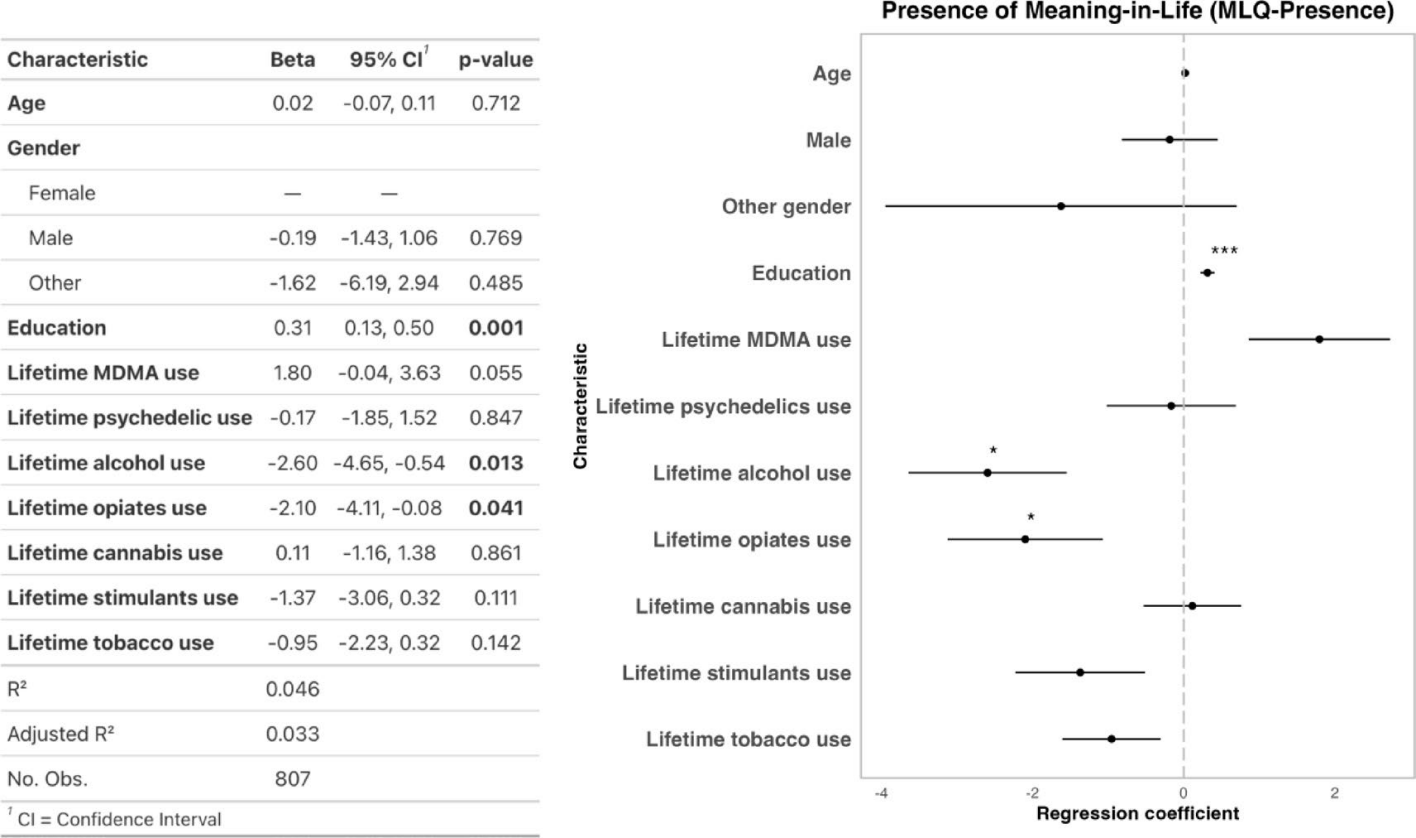
Left: Regression outputs for linear regression model assessing associations between lifetime MDMA use and MLQ-presence scores, with adjustments for age, gender, education, and lifetime use of psychedelics, alcohol, opiates, cannabis, stimulants, and tobacco. Right: Visualization of regression coefficients from the model on the left. * *p* < 0.05 ** *p* < 0.01 ***p < 0.001.

A multiple linear regression model adjusting for the covariates of age, gender, education, and lifetime use of tobacco, cannabis, alcohol, opiates, psychedelics, and stimulants revealed a non-significant association between lifetime MDMA use and MLQ-presence (β = 1.80, 95% CI [-0.04,3.63], *p* = 0.055) (Fig. 2). In addition, a significant negative association was observed between lifetime alcohol use (β=-2.60, 95% CI [-4.65,-0.54], *p* = 0.013) and lifetime opiates use (β=-2.10, 95% CI [-4.11,-0.08], *p* = 0.041) with MLQ-Presence. No significant associations were observed for lifetime stimulants use (β=-1.37, 95% CI [-3.06,0.32], *p* = 0.111), lifetime tobacco use (β=-0.95, 95% CI [-2.23,0.32], *p* = 0.142), lifetime psychedelic use (β=-0.17, 95% CI [-1.85,1.52], *p* = 0.847), or lifetime cannabis use (β = 0.11, 95% CI [-1.16,1.38], *p* = 0.861). For estimated marginal means and detectable effect sizes (Cohen’s d), see supplemental materials (Tables S2-S3). For checks of normality of residuals, multicollinearity, homoscedasticity, and influential points, see supplemental materials (Figures S2-S3). When a listwise deletion approach was used for missing education data, similar trends were observed (Figure S4).

### Hypothesis 2: meaning in life, lifetime MDMA use, and childhood trauma

**Fig. 3:**
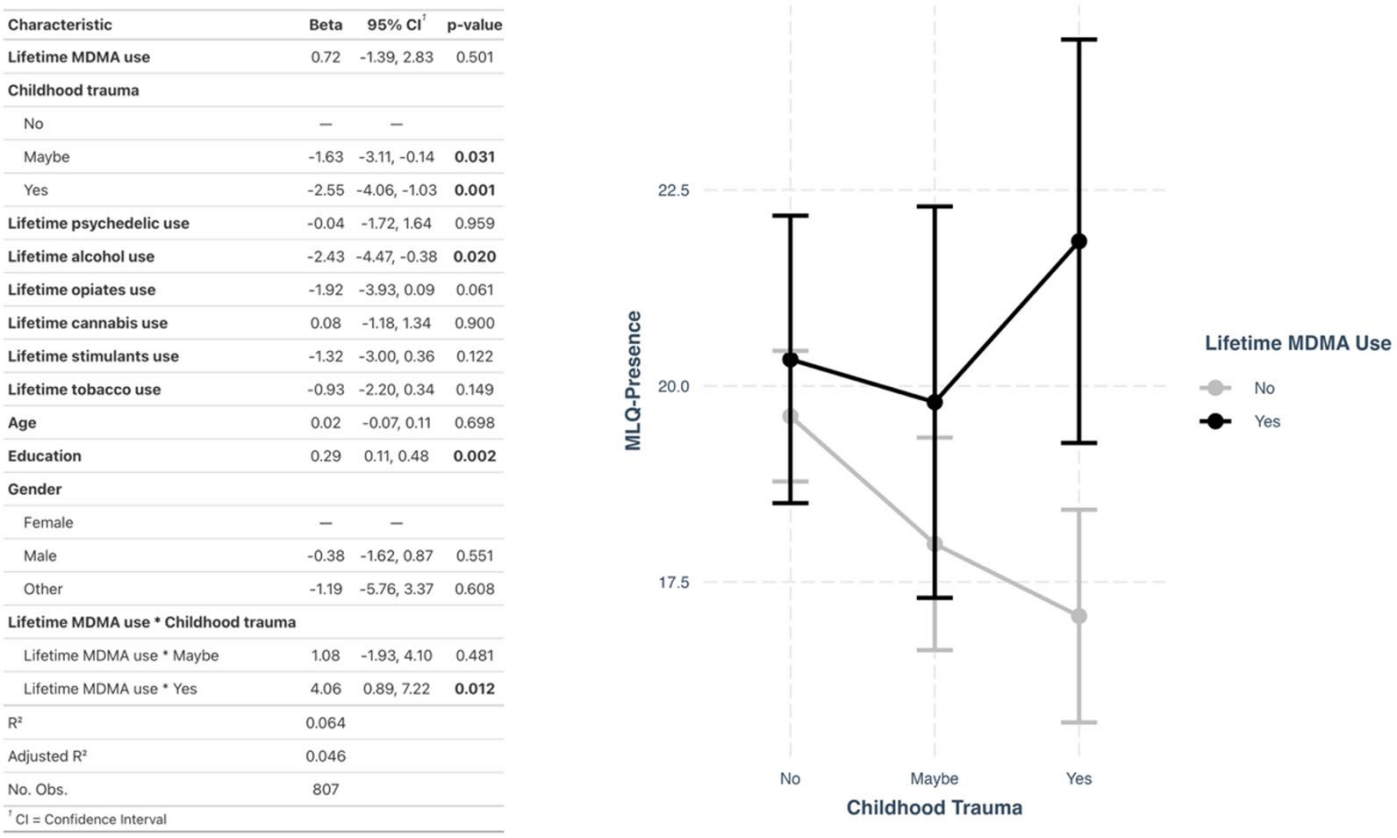
Interactions between MDMA use and childhood trauma on meaning in life. Left: Regression model outputs for the interaction model assessing moderating effects of MDMA use on associations between childhood trauma (‘No’, ‘Maybe’, ‘Yes’) and MLQ-presence. Right: Interaction plot visualizing the model on the left. Bars represent standard error.

Our second hypothesis was that those with childhood trauma would show a stronger association between lifetime use of MDMA and MLQ-Presence scores. For the main model, childhood trauma was measured using the questionnaire responses to “have you been traumatized as a child?” (‘No’, ‘Maybe’, ‘Yes’). The regression model was adjusted for the same covariates as tested in hypothesis 1 (i.e., age, gender, education, and lifetime use of tobacco, cannabis, alcohol, opiates, psychedelics, and stimulants). A significant interaction association with childhood trauma was found where those who reported they were ‘Yes’ psychologically traumatized as a child, showed significantly higher MLQ-Presence scores, had they also reported lifetime MDMA use (β = 4.06, 95% CI [0.89,7.22], p = 0.012) (Fig. 3). For estimated marginal means see supplemental materials (Table S4). The estimated Cohen’s d for the interaction was 0.56, suggesting a medium detectable effect size (Table S5). A sensitivity analysis wherein the independent variable childhood trauma (‘No’, ‘Maybe’, ‘Yes’) was replaced with ACE history (0 vs. 1+) revealed a similar interaction effect (β = 2.66, 95% CI [0.16,5.16], p = 0.037) (Table S6). See supplemental materials for model performance checks such as normality of residuals, multicollinearity, and influential points (Figures S5-S6). A similar interaction trend was observed when a listwise deletion approach was used for handling of missing data of the education variable (Figure S7).

### Exploratory analyses

**Fig. 4:**
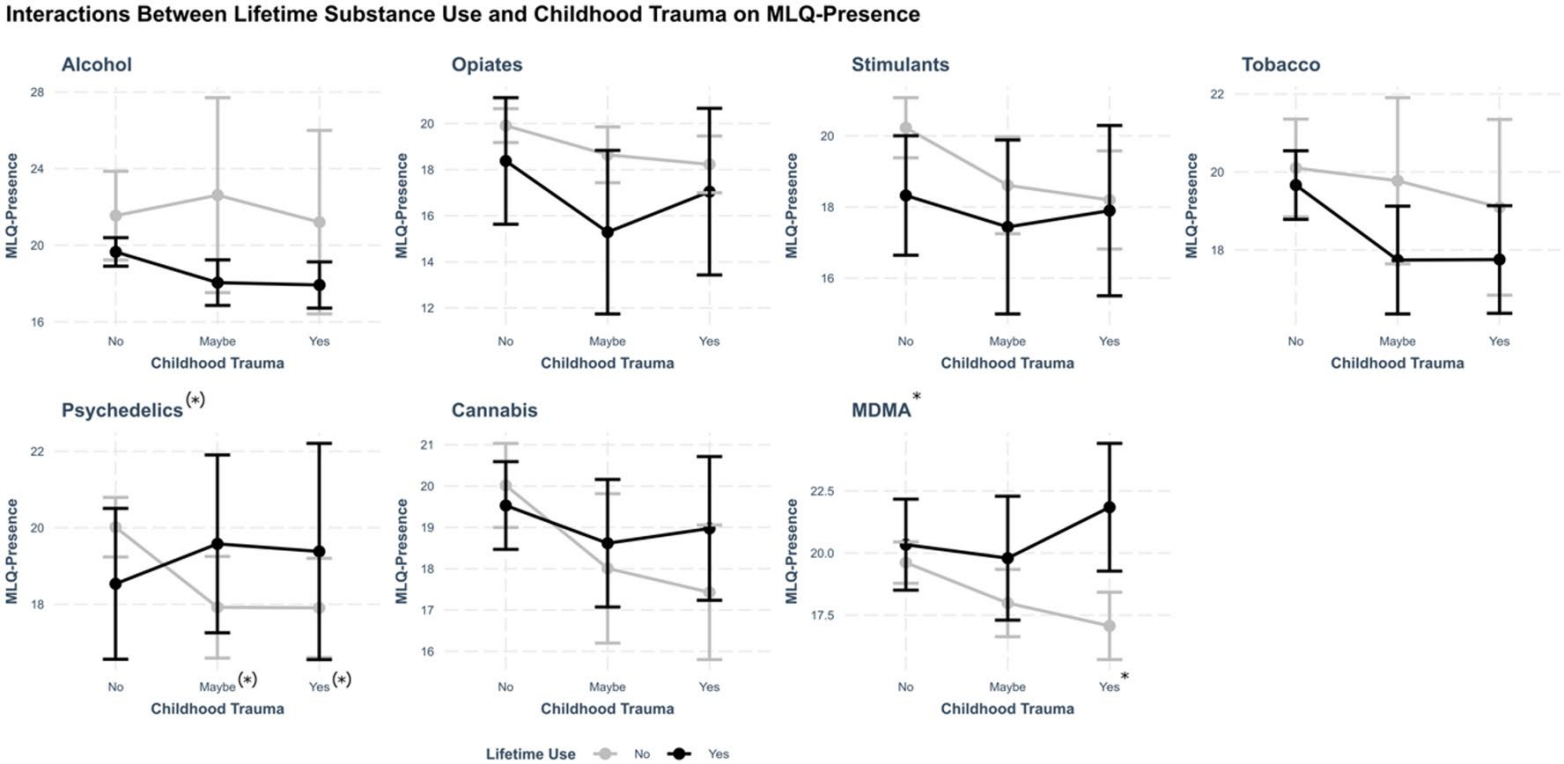
Interactions between substance use and childhood trauma on meaning in life. Interaction effects between lifetime substance use and childhood trauma (‘No’, ‘Maybe’, ‘Yes’) on MLQ-Presence scores. Each figure depicts results from models using childhood trauma as a factor variable. The significance of the interactions is indicated after each substance. The significance of specific levels of trauma is indicated after ‘No’, ‘Maybe’, or ‘Yes’. Significance levels are indicated as follows: (*) *p* < 0.1, * *p* < 0.05.

In addition to the main analyses assessing interactions between lifetime MDMA use and childhood trauma on MLQ-presence, exploratory analyses were conducted to examine whether lifetime use of other substances (alcohol, opiates, cannabis, stimulants, tobacco, and psychedelics) interacted with childhood trauma to predict the presence of meaning in life. As seen in Fig. 4, no interaction terms in the models for other substances were significant, suggesting that the observed interaction effect may be specific to MDMA use. Of note, an interaction between lifetime psychedelic use and a reported history of ‘maybe’ childhood trauma showed a non-significant trend (β = 3.14, 95% CI [0.00,6.28], *p* = 0.050) (Table S7). All models were adjusted for the same covariates as in the main analyses.

## Discussion

The applications of MDMA in conjunction with psychotherapy for trauma-related psychiatric disorders are of growing research interest. However, the range of psychological effects of MDMA and its underlying therapeutic mechanisms has yet to be fully elucidated. In a sample of 807 Swedish adults, the present study aimed to investigate potential associations between lifetime MDMA use and an underexplored psychological factor – meaning in life. Our primary finding was an association between lifetime MDMA use and higher meaning in life among individuals with a history of childhood trauma. Analyses survived adjustment for other substance use and common sociodemographic confounders such as gender, education, and age. Given the growing body of research demonstrating psychotherapeutic benefits of MDMA-assisted psychotherapy for PTSD, our chief finding suggesting an association between MDMA use and positive psychological health in the context of trauma seems especially relevant^4^. However, it should be noted early on that, given the cross-sectional nature of the data, these findings do not imply a causal relationship. Experimental or longitudinal studies are needed to establish whether MDMA use directly contributes to increases in meaning in life or psychological well-being over time.

In contrast to our first hypothesis, the association between lifetime MDMA use and meaning in life did not reach statistical significance (*p* = 0.055). However, the positive direction of this association may still have theoretical relevance. In comparison to lifetime MDMA use, significant negative associations were observed between lifetime use of alcohol and opiates and meaning in life. This aligns with previous literature, which has demonstrated associations between low meaning in life and alcohol dependence, as well as the understanding that alcohol and opiates are considered high-risk substances with no recognized psychotherapeutic applications^65,66^. Thus, these findings may suggest that the observed positive, albeit non-significant, association between MDMA use and meaning in life may still be relevant and substance specific. Moreover, this finding may align with previous observational studies, which have demonstrated associations between naturalistic MDMA use and other positive mental health outcomes, such as lower risk of depression and reduced risk of developing PTSD symptoms^49,67^. Emerging research also suggests that people who have used MDMA report perceived long-term benefits of MDMA use, such as a heightened appreciation of aesthetic experiences, deepened social connection, and positive changes in worldview^47,48^. While these studies have not directly assessed meaning in life, the reported outcomes are also well-established correlates of meaning in life^19,68^. 

The second main finding was a significant positive association between lifetime MDMA use and meaning in life only amongst adults with a reported history of childhood trauma. This finding may be understood within the context of neurobiological theories of MDMA. On an information processing level, the ‘Relaxed Beliefs Under Psychedelics’ (REBUS) model suggests that psychedelics and MDMA relax the precision of high-level priors, thereby facilitating the intrinsic bottom-up flow of information within the system and dramatic transformation of beliefs of the world and self^69^. Ultimately, this relaxation may enable alteration of dysfunctional models and maladaptive core beliefs that often arise from trauma. Previous research has also used predictive coding theories to explain PTSD, attributing various symptoms as a mismatch (or error signal) between the psychological state in the aftermath of a trauma and a desired state^70,71^. It has been suggested that such error signals promote the quest for novel high-level models that can neutralize them, both in healthy and clinical populations^72^. Similarly, the heightened search for meaning in life among childhood trauma survivors observed in our study may mirror such a mismatch (or an error signal) between the psychologically traumatized low meaning state and a desired higher meaning state (Fig. 1). Given our results, these theories may converge to understand MDMA as a catalyst of change toward a more desired sense of meaning in life, thereby reducing global error signaling and attenuating PTSD symptomatology. 

While it is important to remember that the present findings do not implicate a causal relationship between MDMA and meaning making, the association may be relevant in ongoing discussions of MDMA’s clinical applications. MDMA-AT is being increasingly studied as a treatment for trauma-related psychopathologies, but the mechanisms underlying its potential efficacy are not fully understood^13–16^. The present findings may support further investigation into the potential role of meaning making in the therapeutic effects of MDMA-AT for recovery from psychological trauma. This may also be in line with prior qualitative studies, which have found participants from MDMA-AT trials describe positive psychological changes beyond PTSD symptom reduction, such as greater meaning in life, improved interpersonal relationships, and heightened self-awareness^29,30^. The present study, with a considerably larger sample size, bolsters the case for further investigation of meaning-related outcomes associated with MDMA-assisted psychotherapy.

Future research interested in investigating the potential relationship between MDMA and meaning in life may consider taking inspiration from existing naturalistic, clinical, and theoretical research on classical psychedelics. For instance, a recent study used the MLQ to assess changes in meaning in life before and after psychedelic use across various naturalistic and clinical contexts^38^. Similarly, a systematic review on psychedelics and meaning in life highlighted the use of comparable validated questionnaires such as the Purpose in Life Scale (PILS)^37^. Lastly, scholarly discussion has proposed ‘meaning-enhancing properties’ of psychedelics as an important theoretical framework for understanding the psychotherapeutic potential of psychedelics^31–33^. Taken together, it may be relevant to employ similar empirical and theoretical considerations in the study of the relationship between MDMA and meaning in life.

### Limitations

A considerable limitation of this study is its cross-sectional design, which precludes any conclusions about the directionality or causality of the associations observed. A reasonable temporal assumption is that childhood trauma likely preceded MDMA use. Reports from the European Union Drug Agency (EUDA) public health investigation of substance use in Europe support this, as they show that MDMA use typically does not begin until late adolescence or early adulthood^73^. However, the temporal relationship between MDMA use and enhanced meaning in life cannot be inferred from the present study design. Thus, the observed association may have alternative explanations. For instance, individuals with trauma histories who already have higher meaning in life may be more likely to use MDMA. Simultaneously, individuals with trauma and low meaning may be less likely to engage with MDMA use due to concerns about limited mental health resources. However, it is also possible that individuals with trauma and lower meaning in life are more likely to use MDMA due to coping or therapeutic motivations^74^. Another potential reason for differences in meaning in life between those with childhood trauma who have and have not used MDMA may be explained by participant engagement in online networks discussing substance use. Involvement in such networks may result in social support that raises meaning in life independently of substance use. As such, future studies may consider employing qualitative interviews, longitudinal designs, or experimental approaches to better understand the causality of this relationship.

Second, given the observational nature of the study, there are biases with regard to retrospective reporting, response bias, or unidentified latent variables that may have influenced the outcome of interest. Although the analyses controlled for common confounding factors such as polysubstance use, age, education, and gender – it is possible that another common factor or factors predisposing one to MDMA use may also predispose one to greater meaning in life. Third, as our cohort was oversampled for individuals with higher substance use patterns, these findings may not be generalizable to a larger population. While in our sample the prevalence rate of MDMA use was approximately 21%, the National Swedish Country Drug report estimates the lifetime use of MDMA at 2%^73^. We also targeted individuals specifically involved in groups related to substance use and psychedelics, posing a possibility that the observed association is driven by specific behavioral or psychological patterns in communities of this nature. Throughout the naturalistic use of MDMA and psychedelics, there exists a wide range of use that varies in context, motivation of use, and knowledge of drug safety^48^. Past research has demonstrated that Nordic populations who have used psychedelics commonly use for therapeutic or spiritual purposes^75,76^. Our study group may therefore represent a population that is more knowledgeable about drug safety and have specific psychotherapeutic motivations. This may result in a higher representation of MDMA users with positive experiences of MDMA, and in turn overestimate the association between trauma, MDMA use, and meaning in life. Fourth, it is important to note that this survey did not collect any data regarding dose, context of use, or specific experiences related to MDMA or psychedelic use. This can lead to under- or overestimation of effects. The study design is also limited in testing whether MDMA ever caused harm on an individual level, even if no such effects were observed on a population level.

Finally, there exist inherent limitations regarding self-reported data from questionnaires, which can be subject to implicit biases and inaccuracies. As the study of meaning in life is inherently abstract, it presents challenges for empirical measurement and may influence our findings^77^. Although the Meaning in Life Questionnaire is a commonly used assessment of personal purpose and meaning, with studies demonstrating good psychometric properties in over 15 countries, it is important to consider where this instrument lacks^60^. For instance, the statements are specifically designed to be broad in nature, which can lead to varied interpretations amongst participants, with some scholars citing the impact of equating the words “purpose” with “meaning” across cultures^78^.

With regards to our analyses of childhood trauma, we focused on participants’ subjective reports of early psychological trauma. While extensive research has established a strong link between ACEs and adult mental health outcomes, some criticisms point to the oversimplification of the causal relationship between ‘potentially traumatic events’ and the resulting psychological burden^79^. Given that our primary interest lies in the subjective experience of trauma and its influence on global meaning and purpose, we considered perceived psychological trauma to be a more relevant measure for this study. However, this approach has its own limitations. First, individual variations in the interpretation of the term ‘trauma’ may affect participants’ responses, as people understand and define trauma differently. Additionally, retrospective reporting of early life trauma is often unreliable, as trauma can affect memory accuracy^80^. These issues may introduce reporting errors that could impact the observed differences between groups.

## Conclusions

Previous research has indicated therapeutic benefits of MDMA-assisted psychotherapy for the treatment of PTSD. In this study, we provide evidence from a large naturalistic sample that links MDMA use with higher presence of meaning in life in the context of trauma. This association was consistent when controlled for use of other substances, as well as common sociodemographic factors. However, given the high share of MDMA use in this sample, further research is needed to assess the generalizability of these findings. These findings may support further clinical investigation of the therapeutic applications of MDMA for psychological trauma and research that can more readily assess the potential causal relationship between MDMA and meaning in life.

## Supplementary Information

Below is the link to the electronic supplementary material.

Supplementary Material 1

## Data Availability

Given the inclusion of sensitive personal data pertaining to trauma history and illicit substance use, the data was not made publicly available. If interested, the authors can be contacted for the anonymous data. All R scripts used for data analysis in this study can be made available upon request by contacting the corresponding author. Questionnaires can be found in the supplementary materials.
