# Supplementary material for: Lifetime MDMA use and associations with meaning in life in the context of childhood trauma
Source: Sci Rep. 2026 Feb 10;16:5617. doi: 10.1038/s41598-026-37721-6 (PMC12891599; doi:10.1038/s41598-026-37721-6)
Supplement: Supplementary file 1 — Supplementary Material 1 [file 41598_2026_37721_MOESM1_ESM.docx]

**Supplementary Materials for**

Lifetime MDMA Use and Associations with Meaning in Life in the Context of Childhood Trauma

**This PDF file includes:**

Materials

Figures S1 to S7

Tables S1 to S7

**Materials**

*Study announcement I*
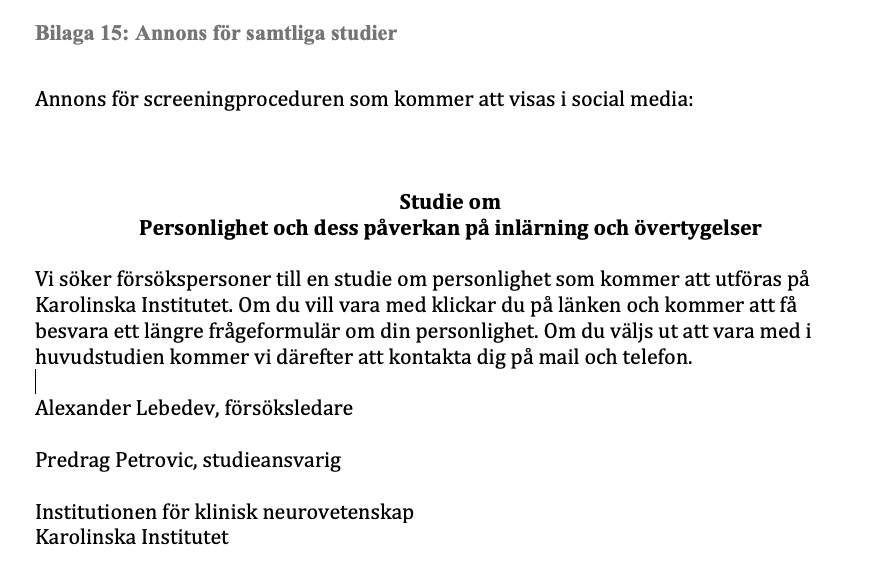


*Relevant questionnaires*

**Childhood_trauma_1** Have you been psychologically traumatized as a child (<17 years)?

- Yes
- Maybe
- No

**Childhood_trauma_2** If yes, choose what the event was from the options below (check all that apply)

- Sexual Abuse
- Physical Abuse
- Neglect
- Bullying
- Household dysfunction (violence or threats of violence among/between family members, substance abuse or mental illness among family members, parental divorce)
- Other

*Substance use questionnaire*

**Substances_Ever** Please specify if you have ever used any of the following drugs:

- Alcohol
- Tobacco
- MDMA / Ecstasy
- Cannabis
- Stimulants (e.g. Amphetamine, Ephedrine, Cocaine)
- Opiates (e.g. Heroin, Morphine, Opium)
- Psychedelics (e.g. LSD, magic mushrooms/psilocybin, Ayahuasca/DMT)
- None

**Psychiatric_diagnoses_1** Have you been diagnosed with any of the following conditions:

- Major depression
- Bipolar disorder
- Schizophrenia
- ADHD
- OCD
- Other

**Psychiatric_diagnoses_2** If ‘Other’, please, specify which

*Meaning in life questionnaire (MLQ)*

|  | Absolutely untrue  (1) | Mostly untrue (2) | | Somewhat untrue  (3) | Can’t say true or false (4) | Somewhat true (5) | Mostly true (6) | Absolutely true (7) |
| --- | --- | --- | --- | --- | --- | --- | --- | --- |
| I am looking for something that makes my life feel meaningful. (1) |  | |  |  |  |  |  |  |
| I am always looking to find my life's purpose. (2) |  | |  |  |  |  |  |  |
| My life has a clear sense of purpose. (3) |  | |  |  |  |  |  |  |
| I have a good sense of what makes my life meaningful. (4) |  | |  |  |  |  |  |  |
| I have discovered a satisfying life purpose. (5) |  | |  |  |  |  |  |  |
| I am always searching for something that makes my life feel significant. (6) |  | |  |  |  |  |  |  |
| I am seeking a purpose or mission for my life.  (7) |  | |  |  |  |  |  |  |
| My life has no clear purpose. (8) |  | |  |  |  |  |  |  |
| I am searching for meaning in my life.  (9) |  | |  |  |  |  |  |  |
| In most ways my life is close to my ideal (10) |  | |  |  |  |  |  |  |

| **Characteristic** | **N = 807**^1^ |
| --- | --- |
| Survey Recruitment Source |  |
| Facebook | 363 (45.0%) |
| Karolinska webpage | 346 (42.9%) |
| Studentkaninen | 7 (0.9%) |
| Forum | 3 (0.4%) |
| Other | 88 (10.9%) |
| ^1^n (%) | |

**Table S1.** **Recruitment source of participants.** Frequencies of recruitment sources for participants. Studentkaninen refers to a Swedish website for recruiting research participants. ‘Other’ refers to participants recruited through word of mouth or referral.


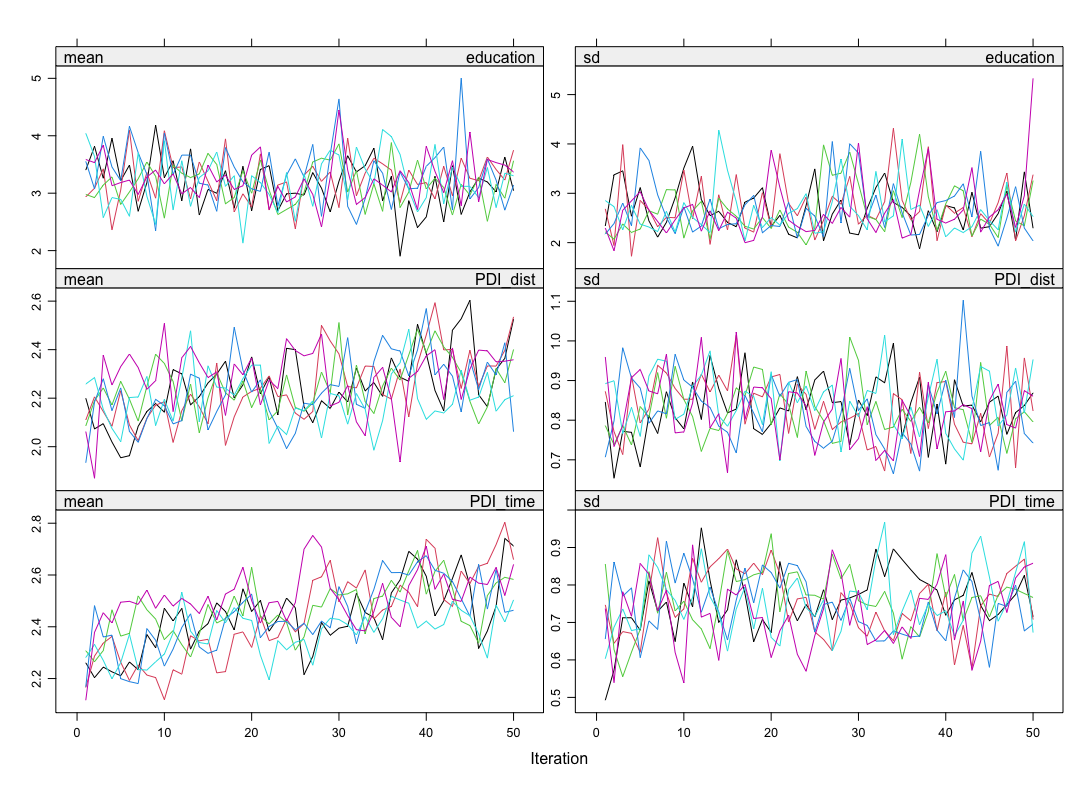


**Figure S1.** **Trace plots**. Checks for convergence after multiple imputation using MICE.

| Variable | Level | EMM | SE | Lower 95% CI | Upper 95% CI |
| --- | --- | --- | --- | --- | --- |
| Gender | Female | 19.65 | 0.72 | 18.25 | 21.06 |
| Gender | Male | 19.47 | 0.78 | 17.94 | 20.99 |
| Gender | Other | 18.03 | 2.40 | 13.32 | 22.74 |
| Education | 3.29727385377943 | 19.05 | 1.01 | 17.07 | 21.03 |
| Lifetime MDMA use | No | 18.15 | 1.12 | 15.95 | 20.35 |
| Lifetime MDMA use | Yes | 19.95 | 1.10 | 17.78 | 22.11 |
| Lifetime psychedelic use | No | 19.13 | 1.05 | 17.07 | 21.19 |
| Lifetime psychedelic use | Yes | 18.97 | 1.14 | 16.72 | 21.21 |
| Lifetime alcohol use | No | 20.35 | 1.31 | 17.77 | 22.92 |
| Lifetime alcohol use | Yes | 17.75 | 0.93 | 15.92 | 19.58 |
| Lifetime opioids use | No | 20.10 | 0.94 | 18.26 | 21.94 |
| Lifetime opiates use | Yes | 18.00 | 1.30 | 15.45 | 20.55 |
| Lifetime cannabis use | No | 18.99 | 1.05 | 16.93 | 21.05 |
| Lifetime cannabis use | Yes | 19.11 | 1.07 | 17.00 | 21.21 |
| Lifetime stimulants use | No | 19.73 | 1.08 | 17.61 | 21.86 |
| Lifetime stimulants use | Yes | 18.36 | 1.11 | 16.18 | 20.54 |
| Lifetime tobacco use | No | 19.53 | 1.07 | 17.42 | 21.63 |
| Lifetime tobacco use | Yes | 18.57 | 1.05 | 16.51 | 20.63 |

**Table S2. Estimated Marginal Means for Model 1.** Corresponds with model from hypothesis 1.

| Variable | β | Cohen's d | Effect Size |
| --- | --- | --- | --- |
| Gender: Male vs Female | -0.19 | -0.03 | Negligible |
| Gender: Other vs Female | -1.62 | -0.22 | Small |
| Lifetime MDMA use | 1.80 | 0.25 | Small |
| Lifetime psychedelic use | -0.17 | -0.02 | Negligible |
| Lifetime alcohol use | -2.60 | -0.36 | Small |
| Lifetime opiates use | -2.10 | -0.29 | Small |
| Lifetime cannabis use | 0.11 | 0.02 | Negligible |
| Lifetime stimulants use | -1.37 | -0.19 | Negligible |
| Lifetime tobacco use | -0.95 | -0.13 | Negligible |

**Table S3. Effect sizes (Cohen’s d) for Model 1.** Corresponds with model from hypothesis 1.


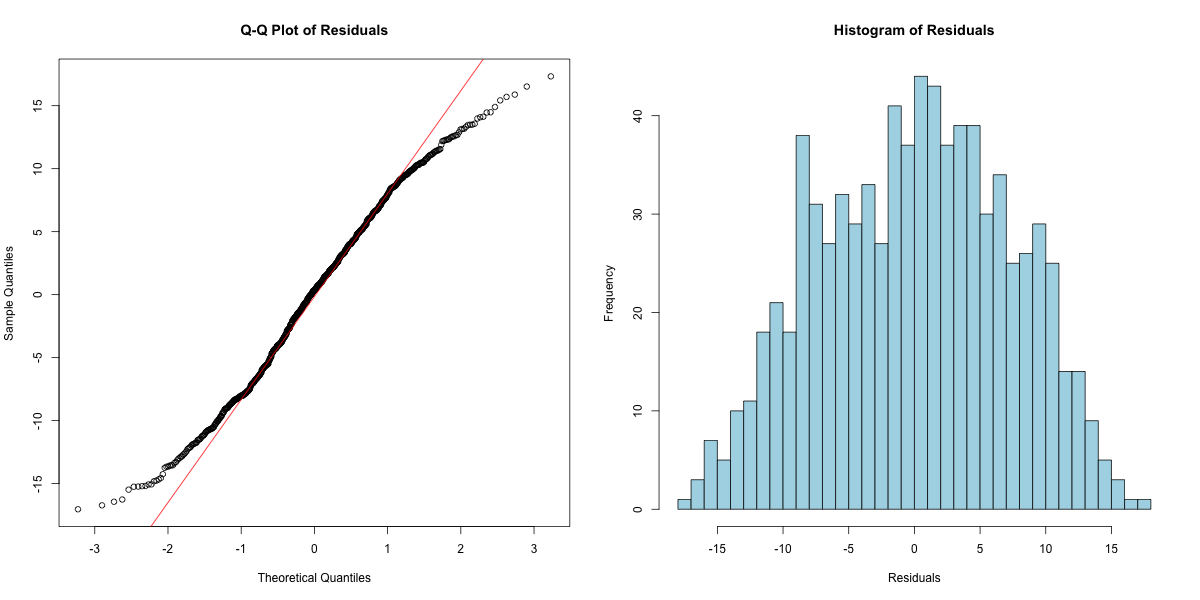


**Figure S2. QQ-plot and Histogram of residuals for Model 1.**


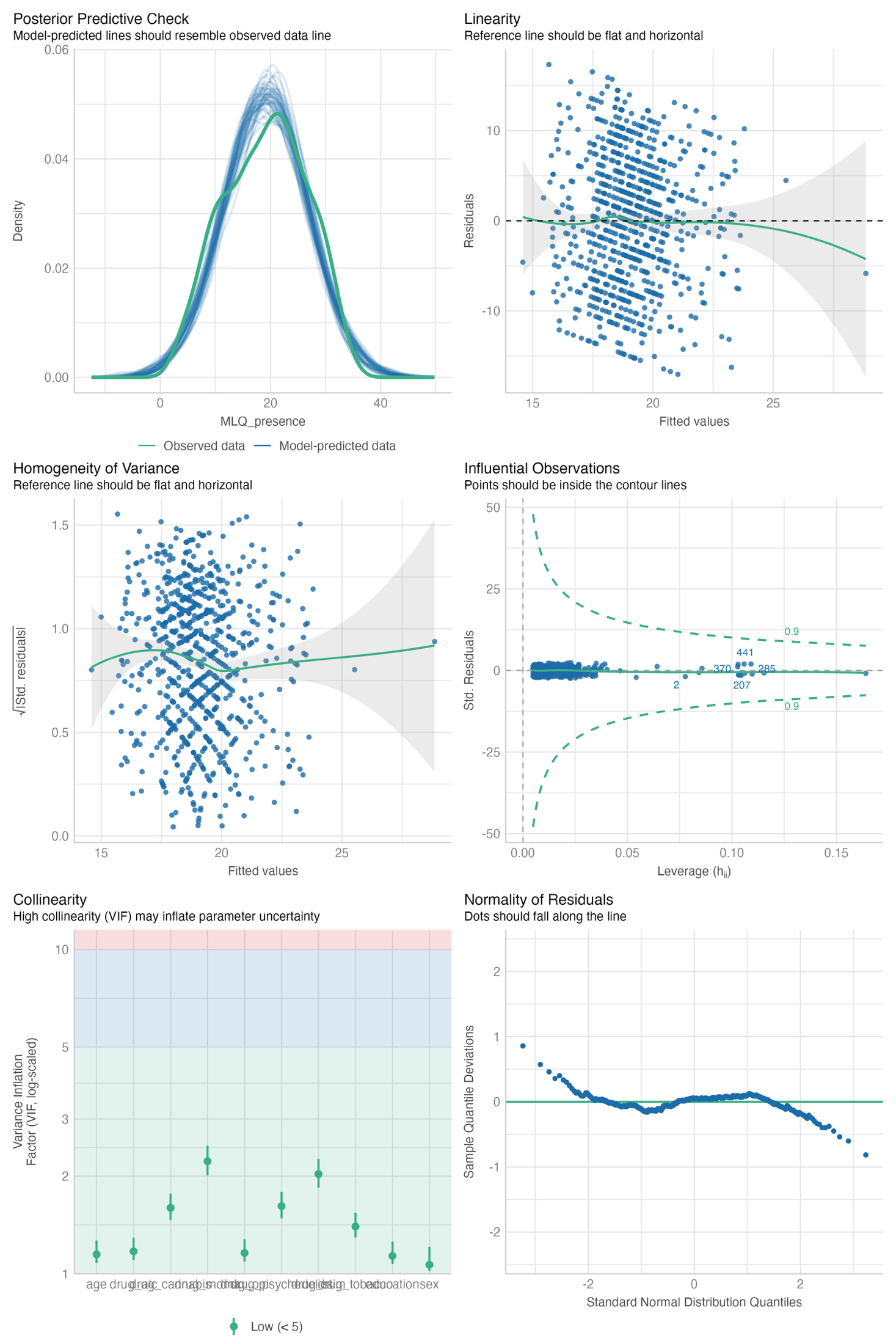


**Figure S3.** **Diagnostics for Model 1**. Figure produced using the R function check_model() from the performance package.


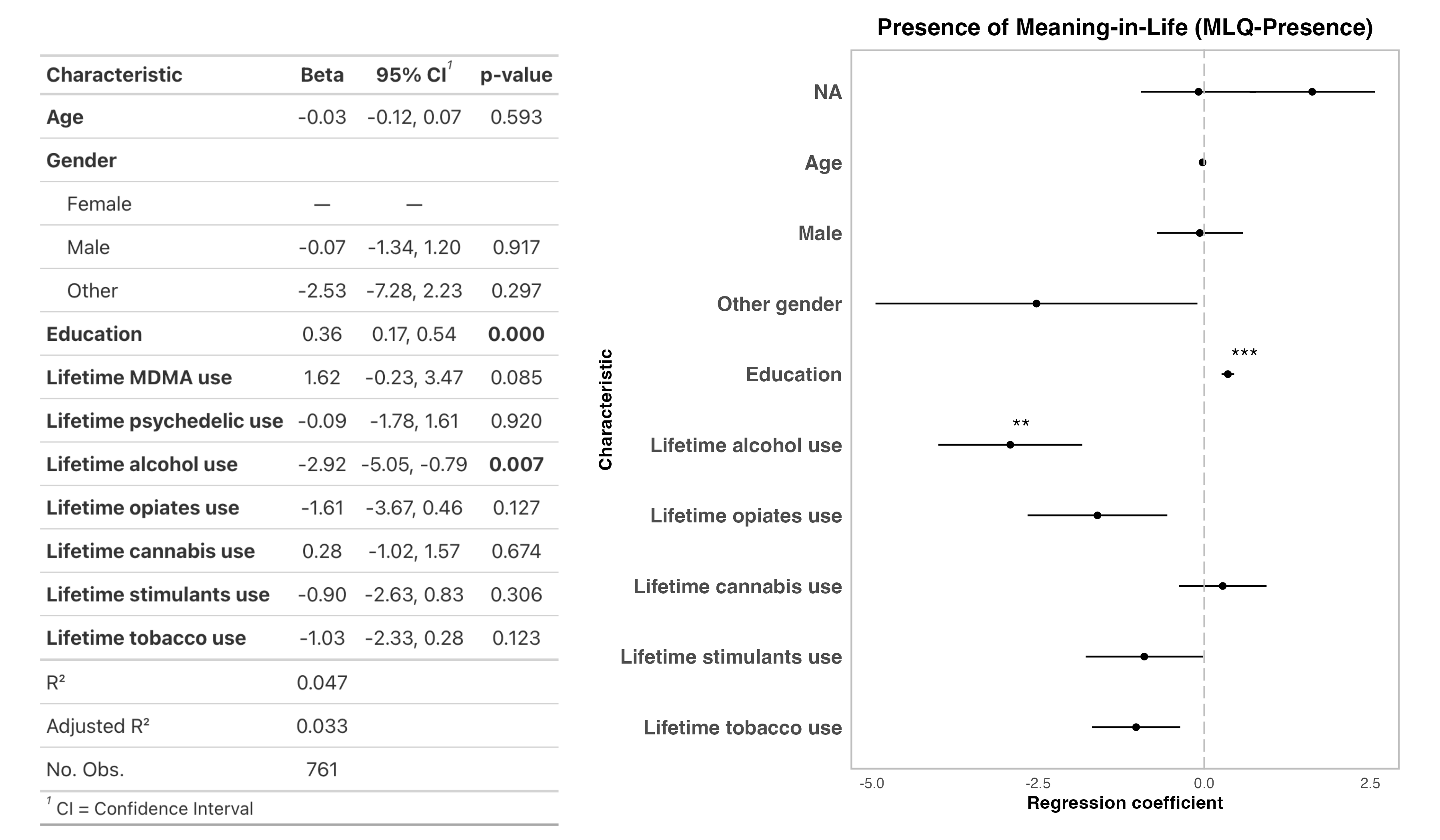


**Figure S4.** **Model 1 Results Using Listwise Deletion.** Sensitivity analysis using listwise deletion approach for missing values rather than MICE.

| Variable | MDMA | ChildhoodTrauma | Level | EMM | SE | Lower 95% CI | Upper 95% CI |
| --- | --- | --- | --- | --- | --- | --- | --- |
| MDMA × Childhood Trauma | No | Yes |  | 19.12 | 1.15 | 16.85 | 21.38 |
| MDMA × Childhood Trauma | Yes | Yes |  | 19.84 | 1.21 | 17.47 | 22.22 |
| MDMA × Childhood Trauma | No | Yes |  | 17.49 | 1.23 | 15.07 | 19.90 |
| MDMA × Childhood Trauma | Yes | Yes |  | 19.30 | 1.45 | 16.45 | 22.14 |
| MDMA × Childhood Trauma | No | Yes |  | 16.57 | 1.28 | 14.05 | 19.08 |
| MDMA × Childhood Trauma | Yes | Yes |  | 21.35 | 1.54 | 18.33 | 24.36 |
| Gender |  |  | Female | 19.47 | 0.72 | 18.05 | 20.88 |
| Gender |  |  | Male | 19.09 | 0.79 | 17.54 | 20.64 |
| Gender |  |  | Other | 18.27 | 2.40 | 13.56 | 22.98 |
| Education |  |  | NA | 18.94 | 1.01 | 16.95 | 20.93 |
| Age |  |  | NA | 18.94 | 1.01 | 16.95 | 20.93 |
| Lifetime psychedelic use |  |  | No | 18.96 | 1.06 | 16.89 | 21.04 |
| Lifetime psychedelic use |  |  | Yes | 18.92 | 1.14 | 16.68 | 21.16 |
| Lifetime alcohol use |  |  | No | 20.16 | 1.31 | 17.58 | 22.73 |
| Lifetime alcohol use |  |  | Yes | 17.73 | 0.93 | 15.90 | 19.56 |
| Lifetime opiates use |  |  | No | 19.90 | 0.95 | 18.04 | 21.76 |
| Lifetime opioids use |  |  | Yes | 17.98 | 1.30 | 15.44 | 20.52 |
| Lifetime cannabis use |  |  | No | 18.90 | 1.05 | 16.84 | 20.97 |
| Lifetime cannabis use |  |  | Yes | 18.98 | 1.07 | 16.87 | 21.09 |
| Lifetime stimulants use |  |  | No | 19.60 | 1.09 | 17.47 | 21.74 |
| Lifetime stimulants use |  |  | Yes | 18.28 | 1.11 | 16.10 | 20.47 |
| Lifetime tobacco use |  |  | No | 19.41 | 1.08 | 17.30 | 21.52 |
| Lifetime tobacco use |  |  | Yes | 18.48 | 1.05 | 16.41 | 20.54 |

**Table S4. Estimated Marginal Means for Model 2.** MDMA x Childhood Trauma refers to the interaction term between lifetime MDMA use (‘Yes’/’No’) and Childhood Trauma (‘No’, ’Maybe’,’ Yes’). Corresponds with results from hypothesis 2.

| Variable | β | Cohen's d | Effect Size |
| --- | --- | --- | --- |
| Gender: Male vs Female | -0.38 | -0.05 | Negligible |
| Gender: Other vs Female | -1.19 | -0.17 | Negligible |
| Lifetime MDMA use | 0.72 | 0.10 | Negligible |
| Lifetime psychedelic use | -0.04 | -0.01 | Negligible |
| Lifetime alcohol use | -2.43 | -0.34 | Small |
| Lifetime opioids use | -1.92 | -0.27 | Small |
| Lifetime cannabis use | 0.08 | 0.01 | Negligible |
| Lifetime stimulants use | -1.32 | -0.18 | Negligible |
| Lifetime tobacco use | -0.93 | -0.13 | Negligible |
| Childhood Trauma: Maybe vs No | -1.63 | -0.23 | Small |
| Childhood Trauma: Yes vs No | -2.55 | -0.35 | Small |
| MDMA x Trauma: Maybe vs No | 1.08 | 0.15 | Negligible |
| MDMA x Trauma: Yes vs No | 4.06 | 0.56 | Medium |

**Table S5. Effect sizes (Cohen’s d) for model 2 (interaction effects).** MDMA x Childhood Trauma refers to the interaction term between lifetime MDMA use (‘Yes’/’No’) and Childhood Trauma (‘No’, ’Maybe’,’ Yes’). Corresponds with results from hypothesis 2.

**
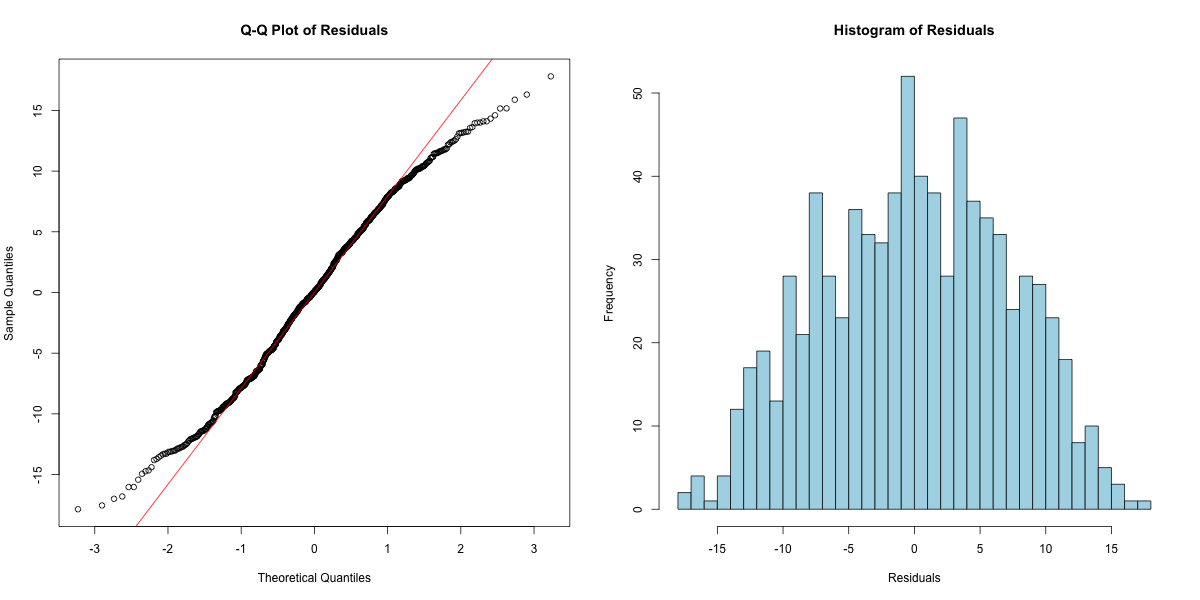
**

**Figure S5.** **QQ-plot and Histogram of Residuals for Model 2.**


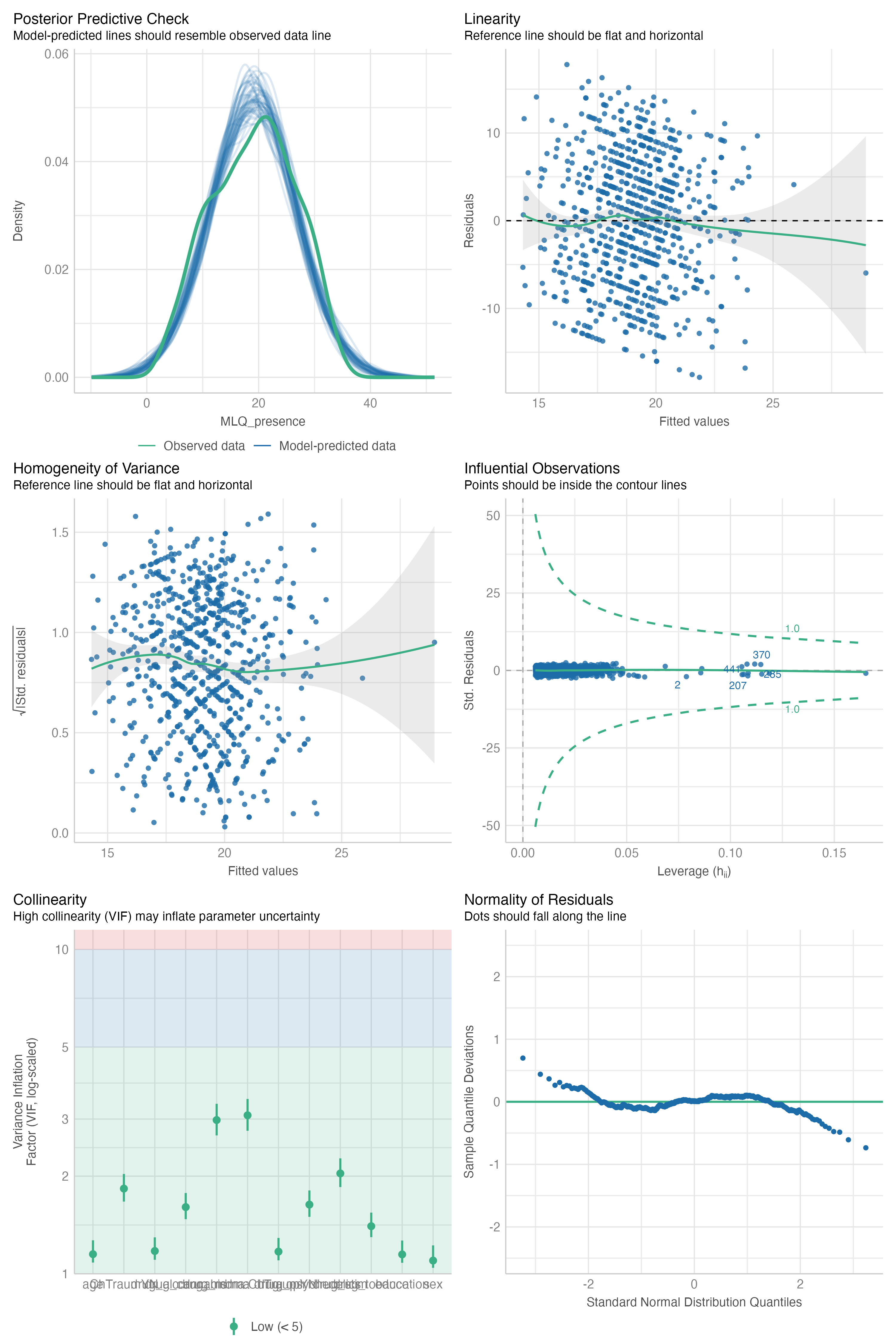


**Figure S6.** **Diagnostics for Model 2**. Figure produced using the R function check_model() from the performance package.


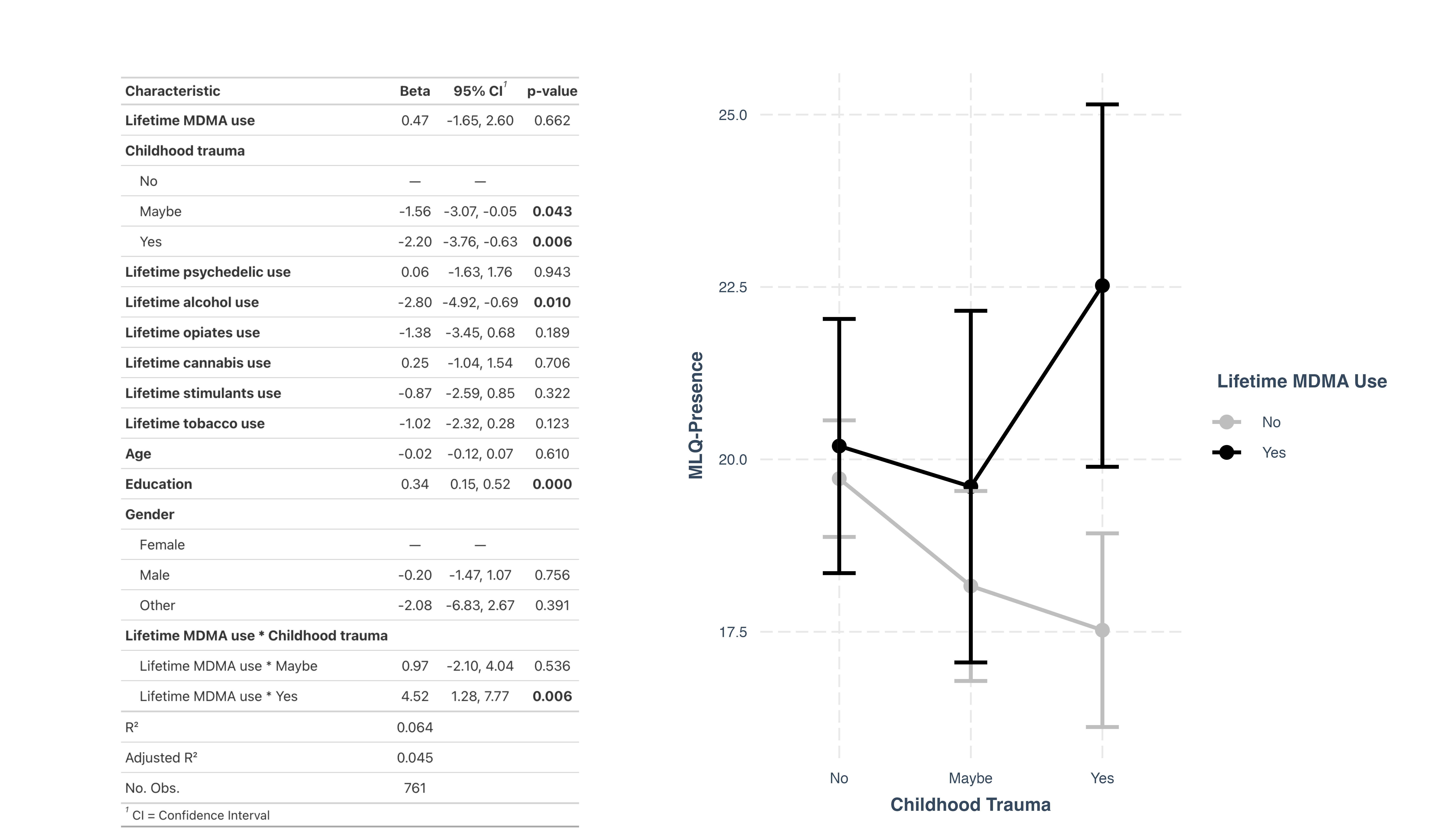


**Figure S7.** **Model 2 Results Using Listwise Deletion.** Sensitivity analysis of model 1 (hypothesis 1) with the listwise deletion approach for missing values rather than MICE.

| **Characteristic** | **Beta** | **95% CI**^1^ | **p-value** |
| --- | --- | --- | --- |
| **Lifetime MDMA use** | 0.72 | -1.39, 2.82 | 0.505 |
| **ACEs** |  |  |  |
| 0 | — | — |  |
| 1+ | -1.75 | -2.93, -0.56 | **0.004** |
| **Lifetime psychedelic use** | -0.10 | -1.78, 1.58 | 0.906 |
| **Lifetime alcohol use** | -2.45 | -4.50, -0.40 | **0.019** |
| **Lifetime opiates use** | -2.06 | -4.07, -0.05 | **0.045** |
| **Lifetime cannabis use** | 0.09 | -1.17, 1.35 | 0.889 |
| **Lifetime stimulants use** | -1.39 | -3.07, 0.29 | 0.104 |
| **Lifetime tobacco use** | -0.92 | -2.19, 0.36 | 0.158 |
| **Age** | 0.02 | -0.07, 0.11 | 0.666 |
| **Education** | 0.29 | 0.11, 0.48 | **0.002** |
| **Gender** |  |  |  |
| Female | — | — |  |
| Male | -0.31 | -1.55, 0.93 | 0.627 |
| Other | -1.05 | -5.61, 3.51 | 0.652 |
| **Lifetime MDMA use * ACEs** |  |  |  |
| Lifetime MDMA use * 1+ | 2.66 | 0.16, 5.16 | **0.037** |
| R² | 0.057 |  |  |
| Adjusted R² | 0.042 |  |  |
| No. Obs. | 807 |  |  |
| ^1^CI = Confidence Interval | | | |

**Table S6. Sensitivity Analysis Using ACE Scores.** Regression outputs for sensitivity analyses assessing interactions between ACEs (0, 1+) and lifetime MDMA use on MLQ-Presence scores. P-values<0.05 are highlighted in bold.

|  | Alcohol*Childhood trauma | Psychedelics*Childhood trauma | Tobacco*Childhood trauma | Stimulants*Childhood trauma | Cannabis*Childhood trauma | Opiates*Childhood trauma | MDMA*Childhood trauma |
| --- | --- | --- | --- | --- | --- | --- | --- |
| (Intercept) | 20.60*** | 21.52*** | 21.04*** | 21.25*** | 21.41*** | 21.17*** | 21.48*** |
|  | [17.42, 23.78] <0.001 | [18.61, 24.43] <0.001 | [18.11, 23.96] <0.001 | [18.35, 24.16] <0.001 | [18.50, 24.33] <0.001 | [18.26, 24.08] <0.001 | [18.57, 24.38] <0.001 |
| Alcohol use | -1.90 | -2.47* | -2.56* | -2.43* | -2.38* | -2.48* | -2.43* |
|  | [-4.28, 0.49] 0.120 | [-4.52, -0.42] 0.018 | [-4.62, -0.51] 0.015 | [-4.49, -0.38] 0.020 | [-4.43, -0.33] 0.023 | [-4.54, -0.43] 0.018 | [-4.47, -0.38] 0.020 |
| Childhood trauma: Maybe vs No | 1.07 | -2.09** | -0.33 | -1.62* | -2.01* | -1.27+ | -1.63* |
|  | [-4.41, 6.55] 0.702 | [-3.57, -0.62] 0.005 | [-2.69, 2.04] 0.786 | [-3.12, -0.11] 0.035 | [-3.95, -0.07] 0.043 | [-2.62, 0.09] 0.067 | [-3.11, -0.14] 0.031 |
| Childhood trauma: Yes vs No | -0.34 | -2.11** | -1.01 | -2.03* | -2.59** | -1.68* | -2.55*** |
|  | [-5.56, 4.88] 0.898 | [-3.58, -0.64] 0.005 | [-3.48, 1.47] 0.424 | [-3.57, -0.49] 0.010 | [-4.42, -0.75] 0.006 | [-3.08, -0.28] 0.019 | [-4.06, -1.03] <0.001 |
| age | 0.02 | 0.01 | 0.02 | 0.02 | 0.02 | 0.02 | 0.02 |
|  | [-0.07, 0.11] 0.622 | [-0.08, 0.11] 0.752 | [-0.07, 0.11] 0.707 | [-0.07, 0.11] 0.643 | [-0.07, 0.11] 0.670 | [-0.07, 0.11] 0.659 | [-0.07, 0.11] 0.698 |
| Male vs Female | -0.31 | -0.45 | -0.33 | -0.33 | -0.36 | -0.29 | -0.38 |
|  | [-1.56, 0.94] 0.624 | [-1.70, 0.80] 0.480 | [-1.58, 0.92] 0.603 | [-1.58, 0.91] 0.601 | [-1.60, 0.89] 0.573 | [-1.54, 0.96] 0.645 | [-1.62, 0.87] 0.551 |
| Other gender vs Female | -1.28 | -0.93 | -1.30 | -1.26 | -1.21 | -1.44 | -1.19 |
|  | [-5.85, 3.29] 0.582 | [-5.50, 3.65] 0.691 | [-5.86, 3.27] 0.578 | [-5.84, 3.32] 0.588 | [-5.79, 3.37] 0.604 | [-6.01, 3.13] 0.536 | [-5.76, 3.37] 0.608 |
| education | 0.30** | 0.31** | 0.30** | 0.29** | 0.30** | 0.30** | 0.29** |
|  | [0.12, 0.48] 0.001 | [0.12, 0.49] 0.001 | [0.11, 0.48] 0.002 | [0.11, 0.48] 0.002 | [0.12, 0.48] 0.001 | [0.11, 0.48] 0.002 | [0.11, 0.48] 0.002 |
| Psychedelics use | 0.04 | -1.48 | 0.03 | 0.04 | 0.03 | 0.04 | -0.04 |
|  | [-1.64, 1.73] 0.961 | [-3.63, 0.66] 0.176 | [-1.66, 1.71] 0.975 | [-1.64, 1.73] 0.960 | [-1.65, 1.71] 0.972 | [-1.64, 1.73] 0.959 | [-1.72, 1.64] 0.959 |
| MDMA use | 1.86* | 1.77+ | 1.89* | 1.81+ | 1.69+ | 1.84* | 0.72 |
|  | [0.03, 3.69] 0.047 | [-0.06, 3.60] 0.058 | [0.06, 3.72] 0.043 | [-0.02, 3.65] 0.053 | [-0.14, 3.53] 0.071 | [0.00, 3.67] 0.050 | [-1.39, 2.83] 0.501 |
| Opiates use | -1.90+ | -2.12* | -1.91+ | -1.95+ | -1.91+ | -1.53 | -1.92+ |
|  | [-3.91, 0.12] 0.065 | [-4.13, -0.10] 0.039 | [-3.93, 0.10] 0.063 | [-3.97, 0.07] 0.059 | [-3.92, 0.11] 0.063 | [-4.34, 1.28] 0.284 | [-3.93, 0.09] 0.061 |
| Cannabis use | 0.13 | 0.15 | 0.11 | 0.15 | -0.49 | 0.11 | 0.08 |
|  | [-1.14, 1.39] 0.845 | [-1.12, 1.41] 0.821 | [-1.16, 1.38] 0.863 | [-1.12, 1.42] 0.817 | [-1.99, 1.01] 0.525 | [-1.16, 1.38] 0.863 | [-1.18, 1.34] 0.900 |
| Stimulants use | -1.43+ | -1.24 | -1.39 | -1.90+ | -1.32 | -1.36 | -1.32 |
|  | [-3.11, 0.26] 0.096 | [-2.92, 0.44] 0.149 | [-3.07, 0.29] 0.104 | [-3.86, 0.05] 0.057 | [-3.01, 0.36] 0.124 | [-3.04, 0.32] 0.114 | [-3.00, 0.36] 0.122 |
| Tobacco use | -0.93 | -0.91 | -0.44 | -0.94 | -0.91 | -0.91 | -0.93 |
|  | [-2.21, 0.34] 0.151 | [-2.18, 0.36] 0.162 | [-1.97, 1.09] 0.574 | [-2.21, 0.34] 0.149 | [-2.18, 0.36] 0.161 | [-2.18, 0.36] 0.162 | [-2.20, 0.34] 0.149 |
| Alcohol use:Childhood trauma: Maybe vs No | -2.67 |  |  |  |  |  |  |
|  | [-8.32, 2.97] 0.353 |  |  |  |  |  |  |
| Alcohol use:Childhood trauma: Yes vs No | -1.38 |  |  |  |  |  |  |
|  | [-6.78, 4.01] 0.615 |  |  |  |  |  |  |
| Psychedelics use:Childhood trauma: Maybe vs No |  | 3.14+ |  |  |  |  |  |
|  |  | [-0.00, 6.28] 0.050 |  |  |  |  |  |
| Psychedelics use:Childhood trauma: Yes vs No |  | 2.96+ |  |  |  |  |  |
|  |  | [-0.55, 6.46] 0.098 |  |  |  |  |  |
| Tobacco use:Childhood trauma: Maybe vs No |  |  | -1.59 |  |  |  |  |
|  |  |  | [-4.41, 1.22] 0.267 |  |  |  |  |
| Tobacco use:Childhood trauma: Yes vs No |  |  | -0.90 |  |  |  |  |
|  |  |  | [-3.82, 2.03] 0.546 |  |  |  |  |
| Stimulants use:Childhood trauma: Maybe vs No |  |  |  | 0.73 |  |  |  |
|  |  |  |  | [-2.24, 3.71] 0.628 |  |  |  |
| Stimulants use:Childhood trauma: Yes vs No |  |  |  | 1.60 |  |  |  |
|  |  |  |  | [-1.44, 4.65] 0.302 |  |  |  |
| Cannabis use:Childhood trauma: Maybe vs No |  |  |  |  | 1.09 |  |  |
|  |  |  |  |  | [-1.51, 3.70] 0.409 |  |  |
| Cannabis use:Childhood trauma: Yes vs No |  |  |  |  | 2.03 |  |  |
|  |  |  |  |  | [-0.62, 4.69] 0.134 |  |  |
| Opiates use:Childhood trauma: Maybe vs No |  |  |  |  |  | -1.82 |  |
|  |  |  |  |  |  | [-6.33, 2.68] 0.427 |  |
| Opiates use:Childhood trauma: Yes vs No |  |  |  |  |  | 0.35 |  |
|  |  |  |  |  |  | [-4.31, 5.01] 0.883 |  |
| MDMA use:Childhood trauma: Maybe vs No |  |  |  |  |  |  | 1.08 |
|  |  |  |  |  |  |  | [-1.93, 4.10] 0.481 |
| MDMA use:Childhood trauma: Yes vs No |  |  |  |  |  |  | 4.06* |
|  |  |  |  |  |  |  | [0.89, 7.22] 0.012 |
| Num.Obs. | 807 | 807 | 807 | 807 | 807 | 807 | 807 |
| R2 | 0.058 | 0.062 | 0.058 | 0.058 | 0.059 | 0.057 | 0.064 |
| R2 Adj. | 0.040 | 0.044 | 0.040 | 0.040 | 0.041 | 0.040 | 0.046 |
| AIC | 5503.4 | 5499.4 | 5503.0 | 5503.3 | 5501.9 | 5503.6 | 5498.0 |
| BIC | 5583.2 | 5579.2 | 5582.8 | 5583.1 | 5581.7 | 5583.4 | 5577.8 |
| Log.Lik. | -2734.712 | -2732.691 | -2734.520 | -2734.648 | -2733.956 | -2734.784 | -2731.995 |
| F | 3.220 | 3.501 | 3.246 | 3.229 | 3.325 | 3.210 | 3.598 |
| RMSE | 7.17 | 7.15 | 7.17 | 7.17 | 7.16 | 7.17 | 7.15 |
| - p < 0.1, * p < 0.05, ** p < 0.01, *** p < 0.001 | | | | | | | |

**Table S7. Exploratory Anlayses Model Outputs.** Model summaries comparing interactions between substance use and childhood trauma (‘No’, ‘Maybe’, ‘Yes’) on MLQ-Presence. These results correspond to those visualized in the main Figure 4.
